# Intra-body dynamics of d-serine reflects the origin of kidney diseases

**DOI:** 10.1007/s10157-021-02052-5

**Published:** 2021-03-25

**Authors:** Hiroki Okushima, Yukimasa Iwata, Atsushi Hesaka, Eri Sugimori, Tatsuhiko Ikeda, Maiko Nakane, Masashi Mita, Terumasa Hayashi, Yoshitaka Isaka, Tomonori Kimura

**Affiliations:** 1 Department of Kidney Disease and Hypertension, Osaka General Medical Center, 3-1-56 Bandaihigashi, Sumiyoshi, Osaka, 558-8558 Japan; 2 KAGAMI Project, National Institutes of Biomedical Innovation, Health and Nutrition (NIBIOHN), 7-6-8 Saito-Asagi, Ibaraki, Osaka, 567-0085 Japan; 3 Reverse Translational Research Project, Center for Rare Disease Research, National Institutes of Biomedical Innovation, Health and Nutrition (NIBIOHN), 7-6-8 Saito-Asagi, Ibaraki, Osaka, 567-0085 Japan; 4 Department of Nephrology, Osaka University Graduate School of Medicine, 2-2 Yamadaoka, Suita, Osaka, 565-0871 Japan; 5 KAGAMI Inc., Ibaraki, Osaka, Japan

**Keywords:** d-Serine, Kidney biopsy, Diagnosis, Lupus nephritis, Chronic kidney disease, Biomarker

## Abstract

**Introduction:**

d-Serine, present only in trace amounts in humans, is now recognized as a biomarker of chronic kidney disease (CKD). CKD is heterogeneous in its original kidney diseases, whose diagnoses require kidney biopsy. In this study, we examined whether the intra-body dynamics of d-serine, indexed by its blood and urinary levels, reflects the origin of kidney diseases.

**Methods:**

Patients with six kinds of kidney disease undergoing kidney biopsy were enrolled in a single center. Levels of d- and l-serine were measured using two-dimensional high-performance liquid chromatography. The associations between the origin of kidney diseases and the intra-body dynamics of d-serine were examined using multivariate cluster analyses.

**Results:**

Unlike the non-CKD profile, patients with CKD showed broadly-distributed profiles of intra-body dynamics of d-serine. The plasma level of d-serine plays a key role in the detection of kidney diseases, whereas a combination of plasma and urinary levels of d-serine distinguished the origin of CKD, especially lupus nephritis.

**Conclusion:**

Intra-body dynamics of d-serine have the potential to predict the origin of kidney diseases. Monitoring of d-serine may guide specific treatments for the origin of kidney diseases.

**Supplementary Information:**

The online version contains supplementary material available at 10.1007/s10157-021-02052-5.

## Introduction

Chronic kidney disease (CKD) is a global concern with more than 10 million patients in Japan and 850 million worldwide [1, 2]. CKD, usually defined by a chronic abnormality in kidney structure or function, including glomerular filtration rate (GFR) [3], is heterogeneous in its origin. The origin of CKD includes immunological, diabetic, aging-related, rare and intractable, and unknown causes of the diseases. The prognosis and treatment of CKD varies and depends on the origin, and the pathological examination of the kidney biopsy specimen often provides key information to make a diagnosis and to assess disease activity [4, 5]. Despite being an important procedure, kidney biopsy has a risk and is performed only when its merit surpasses the risk. Therefore, biomarkers that can help to diagnose kidney diseases are under investigation.

d-Serine is now emerging as a biomarker of kidney diseases [6–9]. d-Serine, one of the d-amino acids, is a mirror-imaged enantiomer (chiral body) of serine that is present only in trace amounts in nature unlike abundant l-serine [10, 11]. For the precise measurements of the levels of d-serine in human samples, two-dimensional high-performance liquid chromatography (2D-HPLC) system [12, 13] has been utilized [6–8]. The 2D-HPLC system potentiated the precise measurements of the trace amount of d-serine in human blood [6]. The blood level of d-serine provides key clinical information since it correlates with one of kidney function, GFR [7], and also reflects the prognosis of the kidney in CKD patients [6]. Moreover, urinary excretion of d-serine provides additional information on the detection of kidney diseases [7, 8]. Accordingly, the assessment of intra-body dynamics of d-serine by measuring its level in blood and urine excretion is useful for monitoring kidney function and the disease activity [14].

These facts suggest a hypothesis regarding whether kidney diseases may differently affect these dynamics based on their origin. If this is the case, monitoring intra-body dynamics of d-serine may help to diagnosis the origin of CKD. This study assessed the d-serine dynamics of patients with CKD in whom kidney biopsies were performed and examined the potential of d-serine to determine the origin of kidney diseases.

## Materials and methods

### Study population

We prospectively enrolled consecutive patients undergoing their first kidney biopsy between 2006 and 2016 at the Department of Kidney Disease and Hypertension, Osaka General Medical Center for diagnosis and/or treatment purposes. Biopsy specimens were routinely analyzed using light, immunofluorescence, and electron microscopy procedures. Clinical and pathological diagnoses were established under the consensus of experienced nephrologists and pathologists. We extracted common causes of kidney diseases by referring to the Histological Classification Scheme of Glomerular Diseases issued by the World Health Organization in 1995. The origin of kidney diseases included IgA nephritis (IgAN), minimal change disease (MCD), membranous nephropathy (MN), diabetic nephropathy (DN), hypertensive nephropathy (HT), and lupus nephritis (LN). Ten patients per origin of kidney disease were enrolled in this study from the study cohort. Inclusion criteria for each disease were as follows: IgAN, range of urinary protein between 0.5 and 1.5 g/gCre and eGFR > 60 mL/min/1.73 m^2^; MCD and MN, meeting the criteria of nephrotic syndrome; DN, history of diabetes, urine albumin-to-creatinine ratio > 30 mg/gCre, and eGFR > 30 mL/min/1.73 m^2^; HN, history of hypertension and eGFR > 30 mL/min/1.73 m^2^. eGFR > 60; LN, meeting the criteria of SLE referred from the 2003 International Society of Nephrology (ISN) / Renal Pathology Society (RPS) classification [15]. Exclusion criteria included the following: (i) cases already started with prednisolone and/or immunosuppressants, (ii) cases with kidney biopsies showing less than 10 glomeruli, (iii) cases with acute kidney injury (AKI), and (iv) cases with secondary MN. Clinical demographics, laboratory data, SLE Disease Activity Index (SLE-DAI) in case of LN [16], and plasma and urine samples were collected at the time of kidney biopsy. The plasma and urinary levels of d-serine were measured from the samples before kidney biopsy. Reference data of non-CKD are from a previous report [7]. The study protocol was approved by the Ethical Committees of Osaka General Medical Center (#29-S0606) and NIBIOHN (#236). This study was conducted in compliance with the ethical principles of the Declaration of Helsinki, and all participants gave written informed consent.

### GFR equations and kidney clearance calculation

Estimated GFR (eGFR) was calculated using the Japanese GFR equation based on serum creatinine (eGFR_creat_) [17]:

$$ {\text{eGFR}}_{{{\text{creat}}}} \left( {{\text{mL}}/\text{min} /1.73\,{\text{m}}^{2} } \right) = 194 \times {\text{Cr}}^{ - 1.094} \times {\text{age}}^{ - 0.287} \times 0.739 \, \left( {\text{if female}} \right). $$

Serum and urinary creatinine were measured enzymatically. Spot urinary levels of chiral amino acids were adjusted by that of creatinine. Fractional excretion (FE, %) was calculated from clearance of substrate divided by that of creatinine, as follows:

$$\begin{aligned} {\text{FE Substrate}} & = \frac{{\text{Substrate clearance}}}{{\text{Creatinine clearance}}} = \frac{{{\text{Us}} \times V/{\text{Ps}}}}{{{\text{Ucre}} \times V/{\text{Pcre}}}} \\ & = \frac{{{\text{Us}} \times {\text{Pcre}}}}{{{\text{Ucre}} \times {\text{Ps}}}} \\ \end{aligned}$$

where Us and Ps represent urinary and plasma levels of substrate, respectively. FE is the ratio of a substrate filtered by the kidney glomerular that is excreted in the urine. Low and high FE indicate tubular reabsorption and excretion, respectively.

### Sample preparation

Sample preparation from human plasma and urine was performed as previously described with modification [12, 13]. Briefly, 20-fold volumes of methanol were added to the sample and an aliquot (10 μL of the supernatant obtained from the methanol homogenate) was placed in a shading brown tube and used for NBD derivatization (1.0 μL of the plasma was used for the reaction). After drying the solution under reduced pressure, 20 μL of 200 mM sodium borate buffer (pH 8.0) and 5 μL of fluorescence labeling reagent [40 mM 4-fluoro-7-nitro-2,1,3-benzoxadiazole (NBD-F) in anhydrous MeCN] were added, and then heated at 60 °C for 2 min. An aqueous 0.1% (v/v) TFA solution (75 μL) was added, and 2 μL of the reaction mixture was applied to 2D-HPLC.

### Determination of serine enantiomers by 2D-HPLC

The enantiomers of serine were quantified using the 2D-HPLC platform [12, 13]. Briefly, the NBD-derivatives of the amino acids were separated using a reversed-phase column (Singularity RP column, 1.0 mm i.d. × 50 mm; provided by KAGAMI Inc., Osaka, Japan) with the gradient elution using aqueous mobile phases containing MeCN and formic acid. To separately determine d- and l-serine, the fractions of serine were automatically collected using a multi-loop valve, and transferred to the enantioselective column (Singularity CSP-001S, 1.5 mm i.d. × 75 mm; KAGAMI Inc.). Then d- and l-serine were separated in the second dimension by the enantioselective column. The mobile phases are the mixed solution of MeOH–MeCN containing formic acid, and the fluorescence detection of the NBD-amino acids was carried out at 530 nm with excitation at 470 nm using two photomultiplier tubes. Target peaks were quantified by scaling the standard peak shapes [18].

### Data processing and multivariate analysis

Data were median-centered and log-transformed before multivariate analysis. The association between chiral amino acids and clinical parameters was analyzed using either unsupervised principal component analysis (PCA) or supervised orthogonal partial least squares (OPLS) analysis. PCA geometrically projects complexed, higher-dimensional data onto lower dimensions called principal components. The loading plot visualizes how strongly each variable affects principal components and how each variable correlates with one another. The score value of each observation is plotted on the score plot to demonstrate the clusters of observations. OPLS-DA was performed to examine the discriminable distributions of a variable based on predictive variables. The goodness-of-fit for the OPLS model was evaluated using *R*^2^*Y* and *Q*^2^*Y* values. *R*^2^*Y* represents the fraction of the variance of the examined variable explained by the model, while *Q*^2^*Y* represents the predictive performance of the model. The values of *R*^2^*Y* and *Q*^2^*Y* more than 0.5 indicate a good goodness-of-fit, and more than 20 permutation tests per an OPLS model were performed to validate the model internally. In the development of the model for LN, an unmodifiable factor, sex, was selected, and we removed it from the related analyses.

### Statistics

Continuous variables are presented as medians and ranges or interquartile ranges (IQR). Categorical variables are given as ratios (%) and counts. Continuous variables were compared using one-way ANOVA with Bonferroni’s post hoc test. Categorical variables were compared using Fisher’s exact test. Logistic regression analyses were performed to estimate the predictability of a binary outcome by a variable. Correlations were analyzed using Pearson’s coefficient, and equality among histological classification was analyzed using Kruskal–Wallis. Statistical significance was defined as *P* < 0.05. Statistical analyses were performed using STATA and R.

## Results

### Plasma level of d-serine and the origin of kidney diseases

We examined intra-body dynamics of d-serine in patients with CKD who underwent kidney biopsy. The baseline characteristics are shown in Table 1 and Supplementary Table 1. We enrolled 10 patients per origin of kidney diseases. The origin of diseases included IgA nephritis (IgAN), minimal change disease (MCD), membranous nephropathy (MN), diabetic nephropathy (DN), hypertensive nephropathy (HT), and lupus nephritis (LN). In the study population, the median age was 54 (interquartile range 40–65), the median estimated GFR (eGFR) was 64.0 (47.5–77.9) mL/min/1.73 m^2^, and the median urinary protein level was 2.71 (0.86–7.39) g/g creatinine.

**Table 1:** Baseline characteristics of the participants

Characteristics	*n* = 60
Age, years	54 (40–66)
Male gender, %	63.3 (38)
Body mass index, kg/m^2^	24.6 (21.6–27.1)
Systolic blood pressure, mmHg	135 (120–154)
Diastolic blood pressure, mmHg	82 (73–90)
Serum protein, g/dL	6.2 (5.2–7.0)
Serum albumin, g/dL	2.9 (2.1–4.2)
Serum creatinine, mg/dL	0.98 (0.74–1.14)
eGFR_creat_, mL/min/1.73 m^2^	64.0 (47.5–77.9)
Serum UN, mg/dL	14.0 (12.0–18.0)
Serum Na, mEq/L	139 (138–141)
Serum K, mEq/L	4.1 (3.9–4.4)
Serum Cl, mEq/L	105 (103–107)
ALT, U/L	20 (14–29)
LDH, U/L	223 (184–268)
Urinary protein, g/gCre	2.71 (0.86–7.39)
Urinary NAG, IU/gCre	15.2 (7.3–27.3)
Urinary β2-MG, mg/gCre	0.256 (0.096–1.123)
Hypertension history, %	58.3 (35)
Diabetes history, %	21.7 (13)
Hyperlipidemia history, %	25.0 (15)
Use of RASI, %	36.7 (22)
Use of other antihypertensive drug, %	35.0 (21)
Use of diuretics, %	31.7 (19)

Values are described as median (IQR) or % (count)

*eGFR* estimated glomerular filtration rate, *UN* urea nitrogen, *NAG* *N*-acetyl-β-d-glucosaminidase, *β2-MG* β2-microglobluin, *RASI* renin-angiotensin system inhibitors

**Fig. 1:**
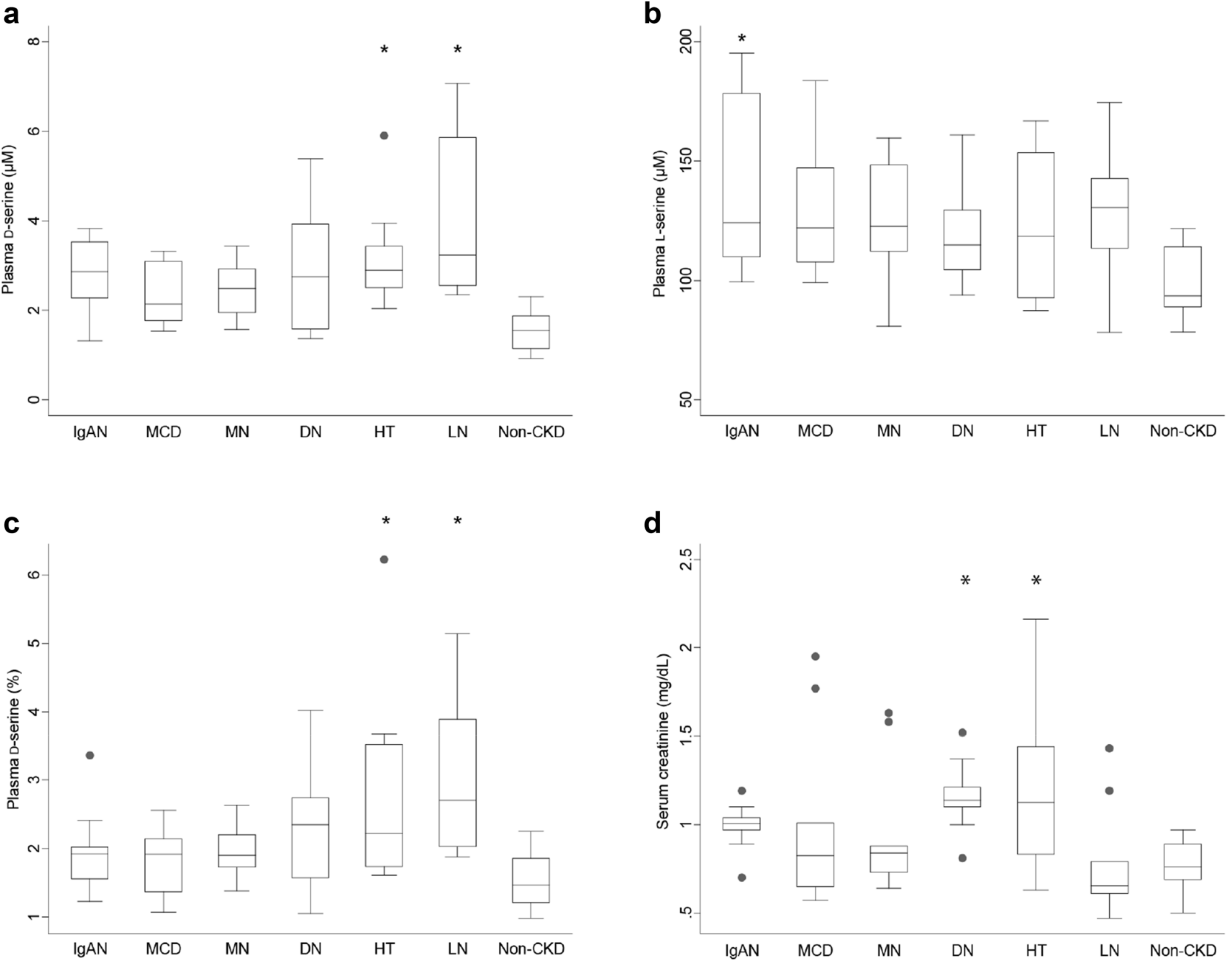
Plasma levels of d-serine in various kidney diseases. Plasma levels of **a** d-serine and **b** l-serine, and **c** plasma ratios of d-serine per total serine in each kidney disease. **d** Serum levels of creatinine in each kidney disease. Reference data of non-chronic kidney disease (CKD) are from [7]. *IgAN* IgA nephritis, *MCD* minimal change disease, *MN* membranous nephropathy, *DN* diabetic nephropathy, *HT* hypertensive nephropathy, *LN* lupus nephritis. *n* = 10 for each disease and 15 for non-CKD. *P < 0.05 versus non-CKD (one-way ANOVA)

We profiled the blood and urinary levels of d-serine in the samples obtained before kidney biopsy. The plasma level of d-serine was quite higher in CKD, and this level was significantly higher in HT and LN than in non-CKD (Fig. 1a). This trend was conserved when the d-serine ratio per total serine was analyzed (Fig. 1b, c). The serum level of creatinine was significantly higher in DN and HT, but not in LN (Fig. 1d), whereas the increase in the plasma level of d-serine was relatively mild in DN (Fig. 1a, c). As expected, the ratio of d-serine over creatinine in blood was uniquely higher in LN than that of non-CKD (Supplementary Fig. 1a). These trends were confirmed in the scatter plots between the plasma levels of d-serine and eGFR (Supplementary Fig. 1b). In patients with DN, eGFR was lower, while the plasma level of d-serine was relatively normal. In patients with HT and LN, on the other hand, the plasma level of d-serine was higher, while eGFR was lower in HT but not in LN. Resultantly, the unique profiles of patients with DN, HT and LN obscured the correlation between the blood level of d-serine and eGFR in the study population [7]. Therefore, the correlation between the blood levels of creatinine and d-serine may variably be affected in the presence of certain types of kidney diseases.

### Urinary excretion of d-serine and the origin of kidney diseases

**Fig. 2:**
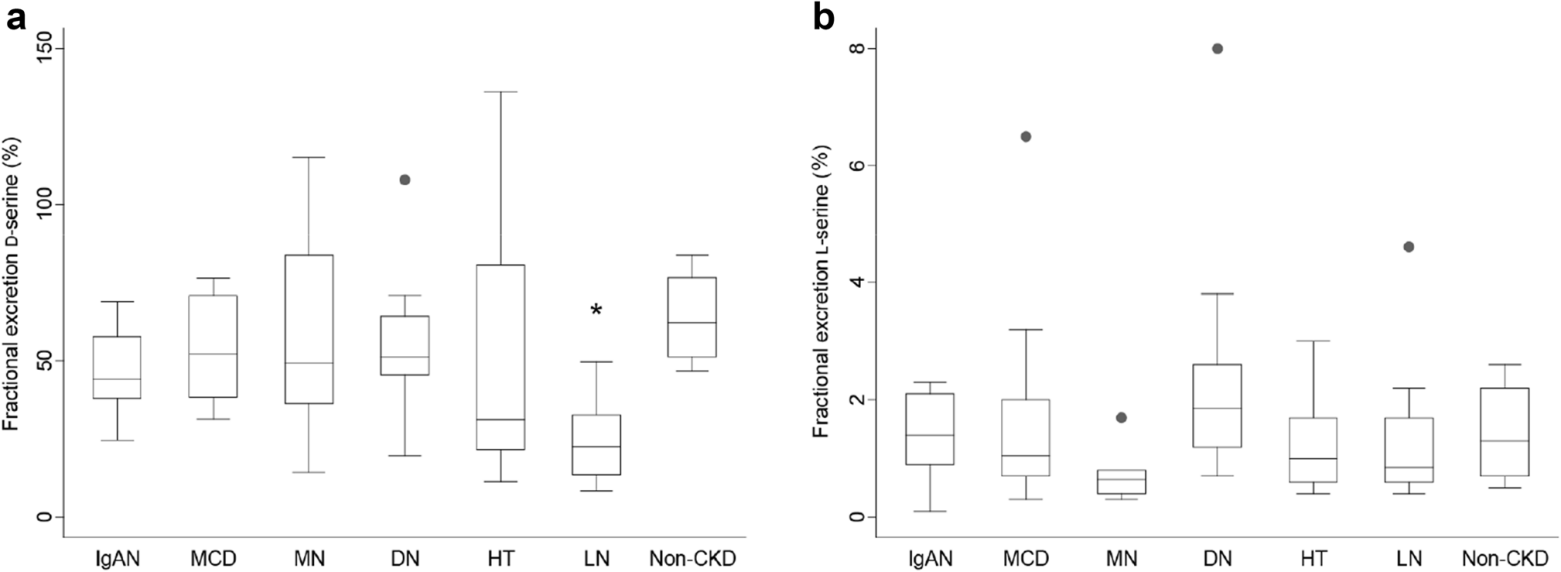
Urinary excretions of d-serine in various kidney diseases. Fractional excretions of **a** d-serine and **b** l-serine in each kidney disease. Reference data of non- chronic kidney disease (CKD) are from [7]. *IgAN* IgA nephritis, *MCD* minimal change disease, *MN* membranous nephropathy, *DN* diabetic nephropathy, *HT* hypertensive nephropathy, *LN* lupus nephritis. *n* = 10 for each disease and 15 for non-CKD. *P < 0.05 versus non-CKD (one-way ANOVA)

We next analyzed fractional excretions (FE) of d-serine (Fig. 2a, b and Supplementary Fig. 2a–c). FE is the ratio of a substrate filtered by the kidney glomeruli that is excreted into the urine. As reported [7], FE of d-serine was much higher than that of l-serine; median values (interquartile ranges) for FE of d- and l-serine were 43.0% (26.9–57.8) and 0.9% (0.6–1.7), respectively (Fig. 2a, b). The range for FE of d-serine was wide, and FE of d-serine was significantly lower in LN than in non-CKD. Thus, FE of d- and l-serine also contains clinical information that reflects the origin of CKD.

### Variances of intra-body dynamics of d-serine and the origin of kidney diseases

**Fig. 3:**
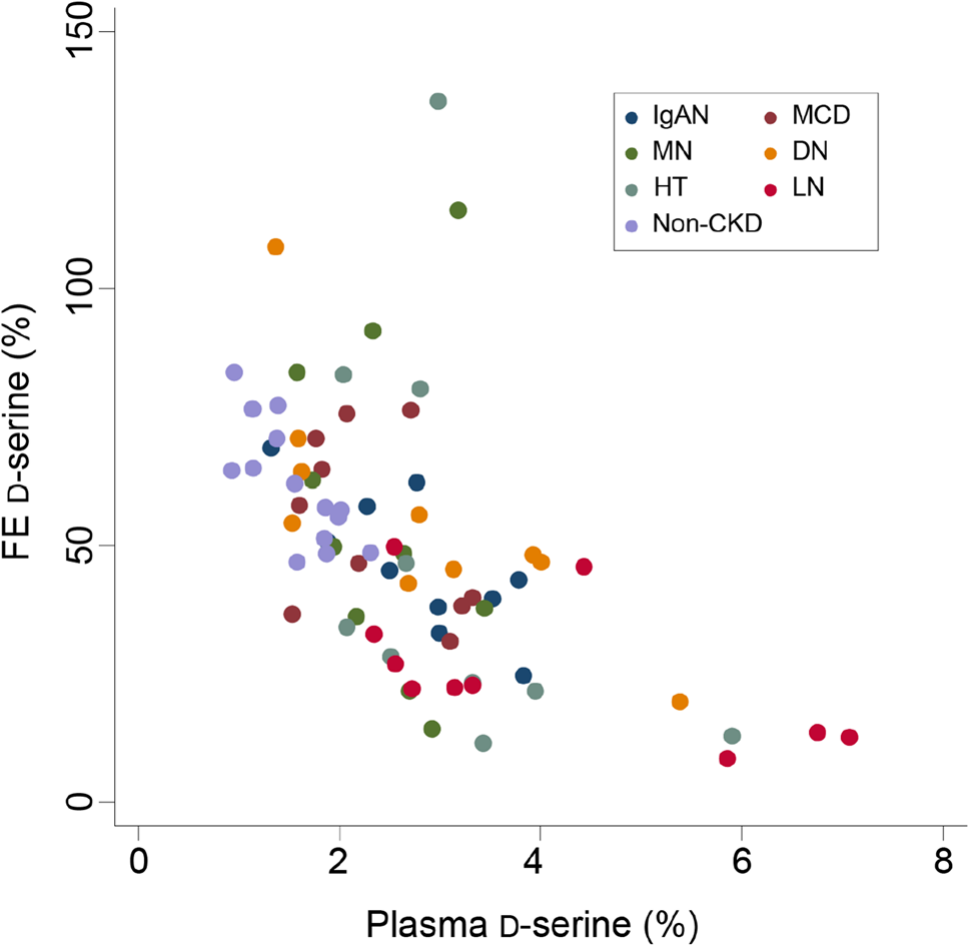
Relation of intra-body dynamics of d-serine and the origin of kidney disease. Relation between plasma levels and fractional excretions of d-serine were plotted. Reference data of non-chronic kidney disease (CKD) are from [7]. *IgAN* IgA nephritis, *MCD* minimal change disease, *MN* membranous nephropathy, *DN* diabetic nephropathy, *HT* hypertensive nephropathy, *LN* lupus nephritis, *FE* fractional excretion. *n* = 10 for each disease and 15 for non-CKD

We analyzed the intra-body dynamics of d-serine, indexed by their plasma levels and FE [7]. Scatter plots of d-serine showed patients with CKD were variably distributed, in contrast to the relatively restricted distribution of non-CKD (Fig. 3). In particular, patients with LN were distributed in the plasma-low FE-low area, a phenomenon seen in the clinical course of a patient with LN [8].

### Association of d-serine profiles and the origin of kidney diseases

**Fig. 4:**
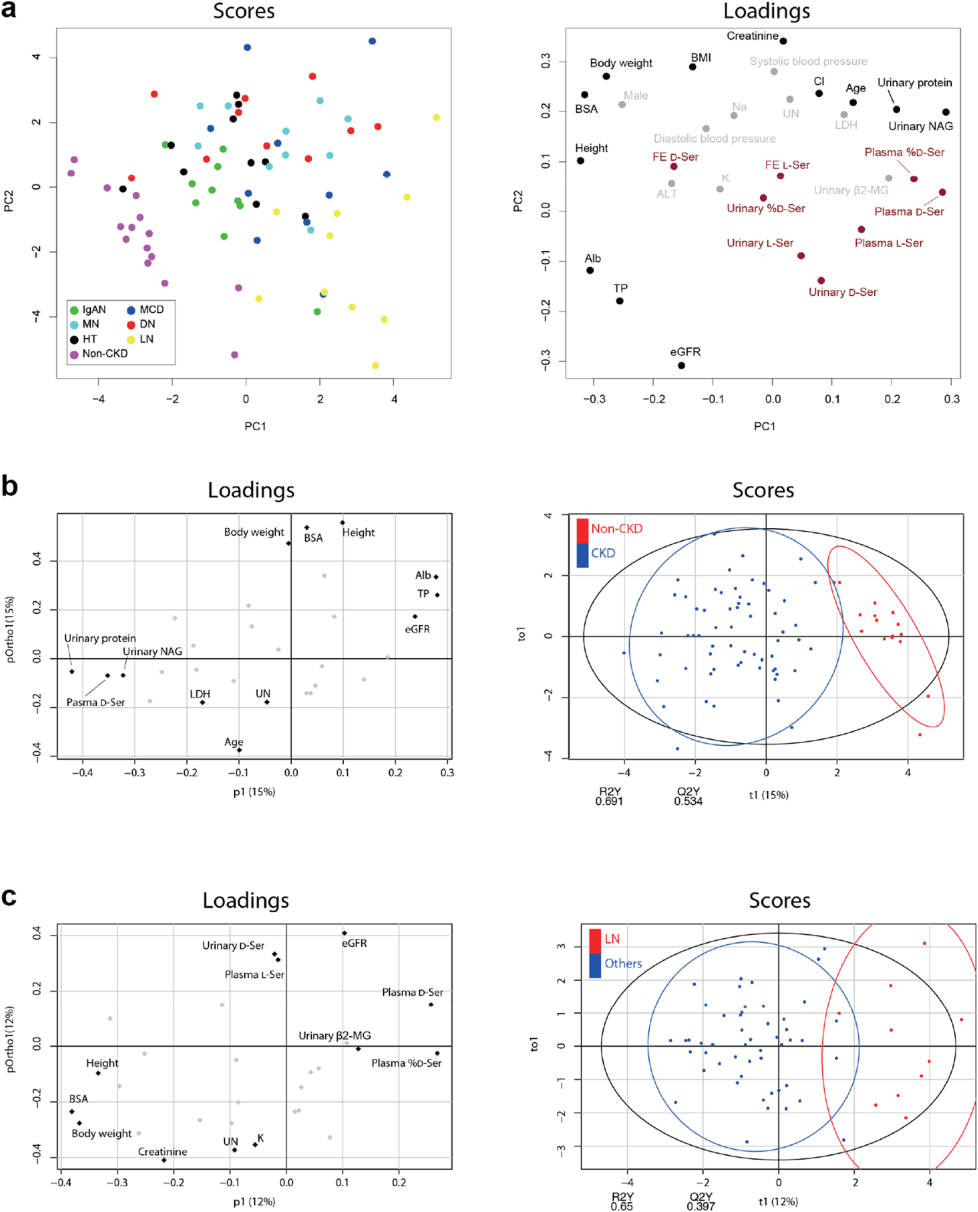
Relation of d-serine profile in the separation of kidney diseases. **a** Primary component analysis (PCA) of d-serine profiles and clinical parameters in associations with original kidney diseases. The score value of each observation is plotted on the score plot to demonstrate the clusters of original kidney diseases. Serine profiles were highlighted in red in the loading plot. **b** Orthogonal projection to latent structure-discriminant analysis (OPLS-DA) loading and score plots derived from d-serine and clinical profiles in chronic kidney disease (CKD) patients compared with non-CKD. **c** OPLS-DA plots in patients wtih lupus nephritis (LN) compared with the rest of study population. *IgAN* IgA nephritis, *MCD* minimal change disease, *MN* membranous nephropathy, *DN* diabetic nephropathy, *HT* hypertensive nephropathy, *FE* fractional excretion, *eGFR* estimated glomerular filtration rate, *UN* urea nitrogen, *NAG* *N*-acetyl-β-d-glucosaminidase, *β2-MG* β2-microglobluin, *BSA* body surface area, *BMI* body mass index, *TP* total protein, *Alb* albumin

To examine the intrinsic variation of d-serine profiles and their relationships with the origin of kidney diseases, we performed principal component analysis (PCA). PCA geometrically projects complexed, higher-dimensional data onto lower dimensions called principal components. Score plots of PCA revealed that patients with non-CKD were clearly distinguished from those with CKD (Fig. 4a). In patients with CKD, the profile of LN was relatively constricted to 3–5 o’clock area, suggesting the presence of a unique profile. d-Serine profiles represented unique characteristics since they distributed dispersedly and apart from classical clinical factors in the loading plots.

To further determine the key parameters for the separation of disease profiles, we performed orthogonal projection to latent structure-discriminant analysis (OPLS-DA). OPLS-DA is a multivariate method used to identify predictive factors to discriminate between groups that separate the variation into target correlating variation (p1) and target non-correlating variation (pOrtho1). Patients with CKD were largely distinguished from non-CKD by factors, such as plasma d-serine, urinary protein and eGFR (Fig. 4b), corroborating the utility of d-serine as a biomarker of kidney diseases (Fig. 1a, c) [7].

We then examined the discriminability of each origin of kidney disease. Patients with LN were largely distinguished from those having other diseases (Fig. 4c). The plasma level of d-serine and the plasma ratio of d-serine are the one of the biggest contributors to this separation, suggesting the key role of d-serine in the differentiation of kidney diseases. Odds ratios for the detection of LN were 2.4 (1.4–4.0) for plasma level of d-serine and 2.5 (1.3–4.7) for its plasma ratio. As a sub-analysis of LN patients, we examined the associations of d-serine profile with clinical and laboratory data that are associated with SLE or reflect its activity, and with histological categories (Supplementary Fig. 3a–c) [15]. In this analysis, higher FE of d-serine was associated with higher level of anti-double-stranded DNA antibody and lower level of C4 in blood, both of which reflect the activity of LN [19]. Urinary excretions of d- and l-serine were also useful for the diagnosis of some diseases. For the separation of DN from other diseases, the urinary ratio of d-serine and FE of l-serine was useful (Supplementary Fig. 4a). Patients with MN were separated from those having other diseases mainly by the urinary level of d-serine and the blood level of protein (Supplementary Figs. 4b and 5). Both plasma and urinary levels of d-serine have shown their potentials to distinguish the various origins of kidney diseases.

## Discussion

In this study, we demonstrated the utility of d-serine monitoring in the diagnosis of the origin of kidney diseases. Intra-body dynamics of d-serine varies depending on the various origins of kidney diseases and has the potential to distinguish the origins of kidney diseases. The results of this study shed a light on the accurate diagnosis of the origin of kidney diseases and may assist in the correct diagnosis of cases that require kidney biopsy. The variable profiles of d-serine in the different kidney diseases take are of great interest as they may help to discover new pathophysiology of kidney diseases.

Patients with LN showed a distinctive profile of d-serine, and the plasma level of d-serine is one of the key factors that has a potential for diagnosis. In the study population, the serum level of creatinine was not significantly high in patients with LN. Because the plasma level of d-serine correlates with the blood level of creatinine [7], the discrepancy seen in LN may be a key phenomenon and reflect direct pathological connections of d-serine in LN. On the other hand, FE of d-serine was decreased and may reflect the activity of disease in LN patients. In patients with CKD, the increase in FE of d-serine precedes the worsening of GFR and the increase of plasma d-serine levels. Conversely, the profile of d-serine changes in reflection of therapy [8]. FE of d-serine transiently increased before the normalization of plasma d-serine levels in a patient with rapidly progressive glomerulonephritis due to LN [8, 14]. Clinical spectrum of LN is broad and ranges from acute to chronic, and FE of d-serine changes dynamically in the time course and the clinical spectrum of LN. A higher level of plasma d-serine with lower urinary excretion, due to either decreased actual GFR (i.e., we could not exclude the potential discrepancy between estimated and actual GFR) or direct effect on the reabsorption of d-serine at proximal tubules, may indicate a unique characteristic of LN and predict a worse prognosis.

Basically, the plasma level of d-serine reflects GFR, whereas this relationship seems to vary depending on the origin of kidney diseases. Increased plasma level of d-serine was also seen in patients with HT (Fig. 1a). In these patients, this increase was in conjunction with the increased the serum level of creatinine, representing the correlation between GFR and the plasma level of d-serine [7]. Urinary excretion of d-serine also has a diagnostic potential for DN and MN. In these patients, urinary excretion of d-serine was variable, though not significant (Supplementary Fig. 2a, c). Of note, FE of l-serine was relatively high in DN and was one of the key factors for diagnosis. Because FE of l-serine is usually well conserved and less affected than that of d-serine by kidney tubular injury [7], an increased FE of l-serine may be an overlooked factor that can distinguish DN. The dynamics of d-serine was balanced by such factors as food intake, synthesis through a racemic reaction in brain [20] and by gut microbiota [21, 22], and urinary excretion [7, 9]. On the other hand, the blood level of d-serine is well regulated by the kidney through the adjustment of urinary excretion, and this feature is the key for the strong correlation with GFR [7, 9]. In the presence of kidney diseases, however, the correlation between d-serine and eGFR became weaker, because of the disturbance in the regulation of d-serine dynamics.

The current study does not underestimate the classical clinical factors. Rather, these factors are selected as key predictors, which validate the quality of the study. For example, in MCD, a disease frequently complicated with heavy proteinuria [23], the urinary protein level is a strong diagnostic factor. The fact that the profiles of intra-body dynamics of d-serine are selected as the predictors for the diagnosis even under the adjustment for the presence of classical clinical factors strongly suggest that d-serine has a strong clinical impact on the diagnosis of the origin of kidney diseases.

The current study has several limitations that need to be recognized for the interpretation of the results. The number of patients for each disease was limited and the prognoses of these patients were not studied, and these features may have obscured further relationships between d-serine and kidney diseases. Because of the limited number of participants, it is necessary to perform sub-group analyses in such diseases as IgAN or DN, in which the spectrum of the diseases is relatively broad. Even with these limitations, this study clearly identified the origin of kidney diseases in which the patients will diagnostically benefit from monitoring d-serine.

In conclusion, the assessment of the intra-body dynamics of d-serine is useful for the diagnosis of original kidney diseases. Monitoring of d-serine may guide specific treatments for the origin of kidney diseases, especially LN. This study opens a new direction for precision medicine using d-serine measurements.

## Supplementary Information

Below is the link to the electronic supplementary material.

Supplementary file1 (PDF 417 kb)
