# Supplementary material for: Intra-body dynamics of d-serine reflects the origin of kidney diseases
Source: Clin Exp Nephrol. 2021 Mar 25;25(8):893–901. doi: 10.1007/s10157-021-02052-5 (PMC8260539; doi:10.1007/s10157-021-02052-5)
Supplement: Supplementary file 1 — Supplementary file1 (PDF 417 kb) [file 10157_2021_2052_MOESM1_ESM.pdf]

Supplementary information

Intra-body dynamics of D-Serine reflect the origin of kidney diseases.

Hiroki Okushima, Yukimasa Iwata, Atsushi Hesaka, Eri Sugimori,
Tatsuhiko Ikeda, Maiko Nakane, Masashi Mita, Terumasa Hayashi,
Yoshitaka Isaka, Tomonori Kimura

5 Supplementary figures
1 Supplementary table

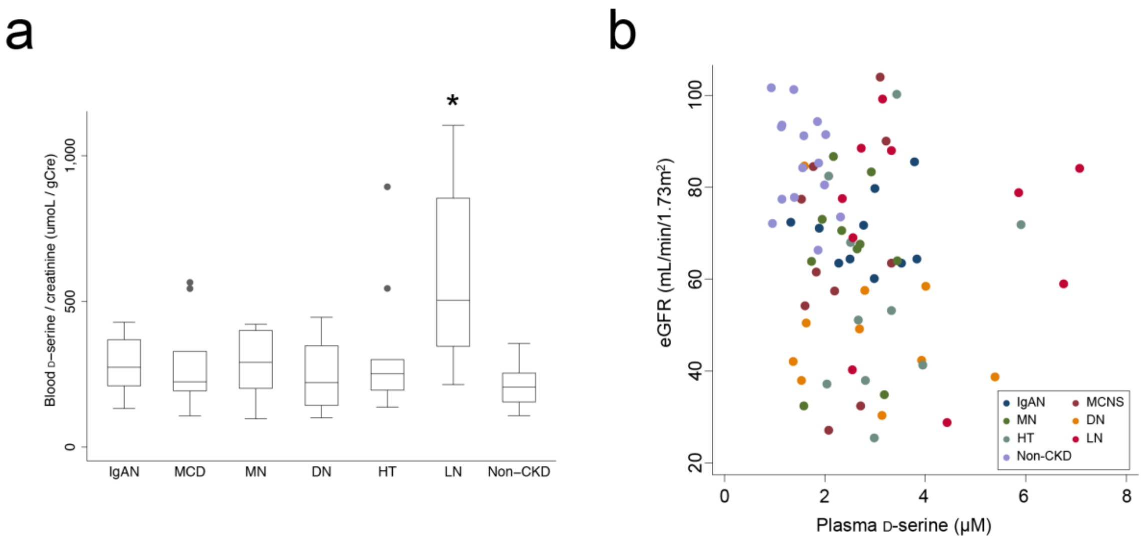

Supplementary Fig. 1 Relation of plasma level of D-serine, serum level of creatinine, and estimated glomerular filtration rate (eGFR). a Blood ratios of D-serine per creatinine in each kidney disease. **b** Relation between eGFR and plasma level of D-serine were plotted. Reference data of non-chronic kidney disease (CKD) are from [7]**.** IgAN, IgA nephritis; MCD, minimal change disease; MN, membranous nephropathy; DN, diabetic nephropathy; HT, hypertensive nephropathy; LN, lupus nephritis. n = 10 for each disease and 15 for non-CKD. *P < 0.05 versus non-CKD (one-way ANOVA).

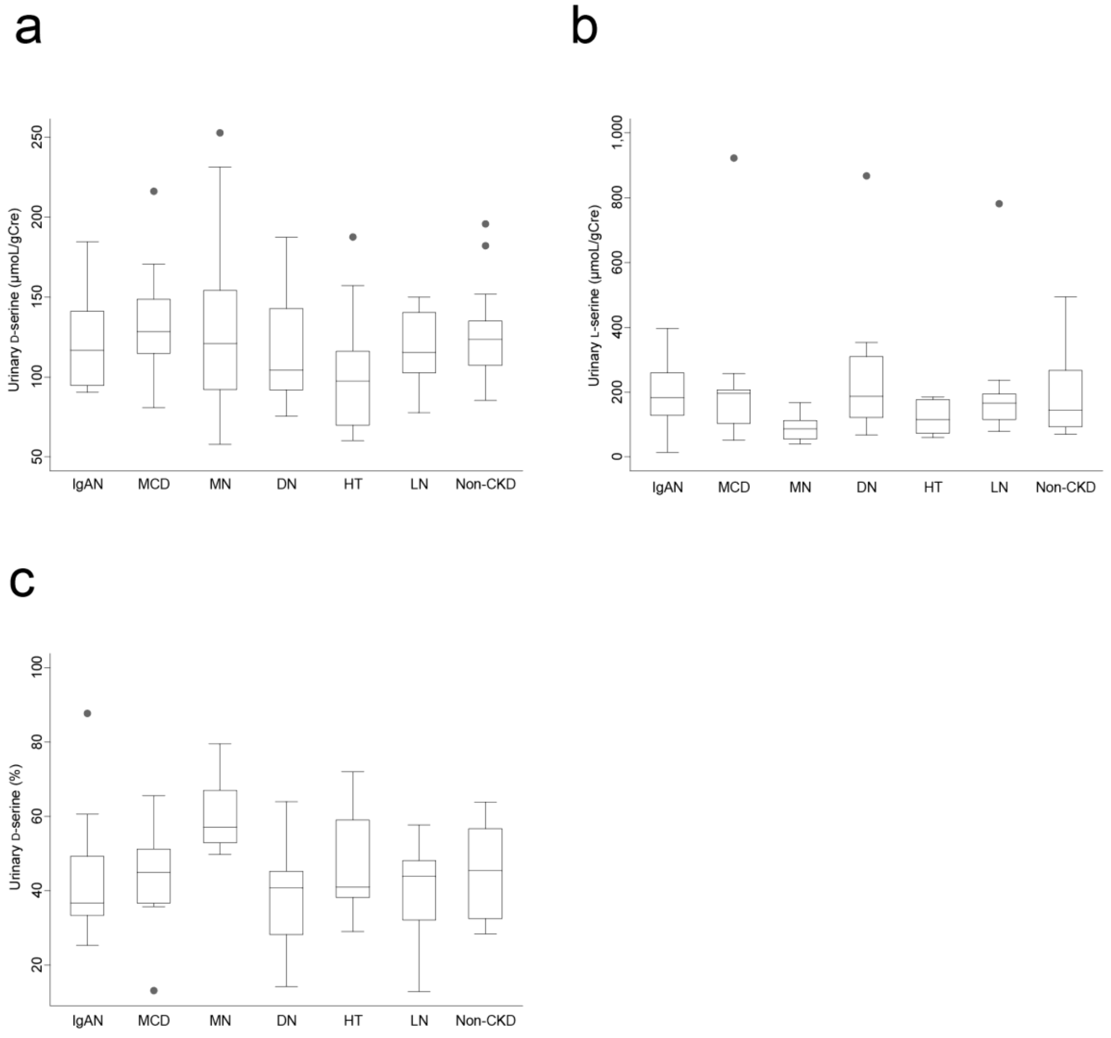

Supplementary Fig. 2 Urinary excretions of D-serine in various kidney diseases. a-c Urinary levels of (**a**) D-serine and (**b**) L-serine, or (**c**) urinary ratio of D-serine per total serine, in each kidney disease. Reference data of non-CKD are from [7]. IgAN, IgA nephritis; MCD, minimal change disease; MN, membranous nephropathy; DN, diabetic nephropathy; HT, hypertensive nephropathy; LN, lupus nephritis; CKD, chronic kidney disease. *n* = 10 for each disease and 15 for non-CKD.

Figure S3

a

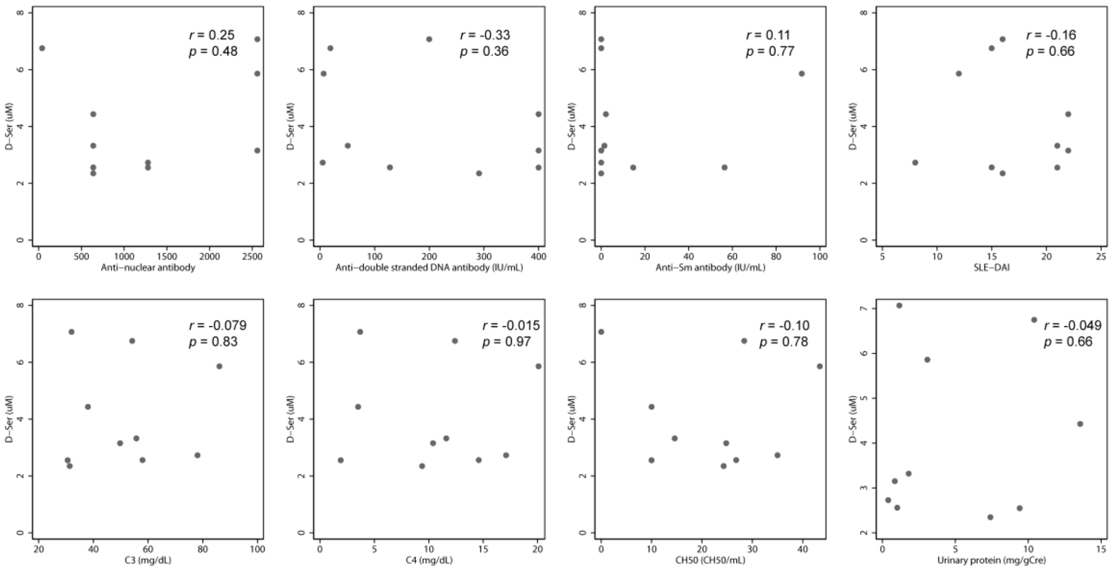

b

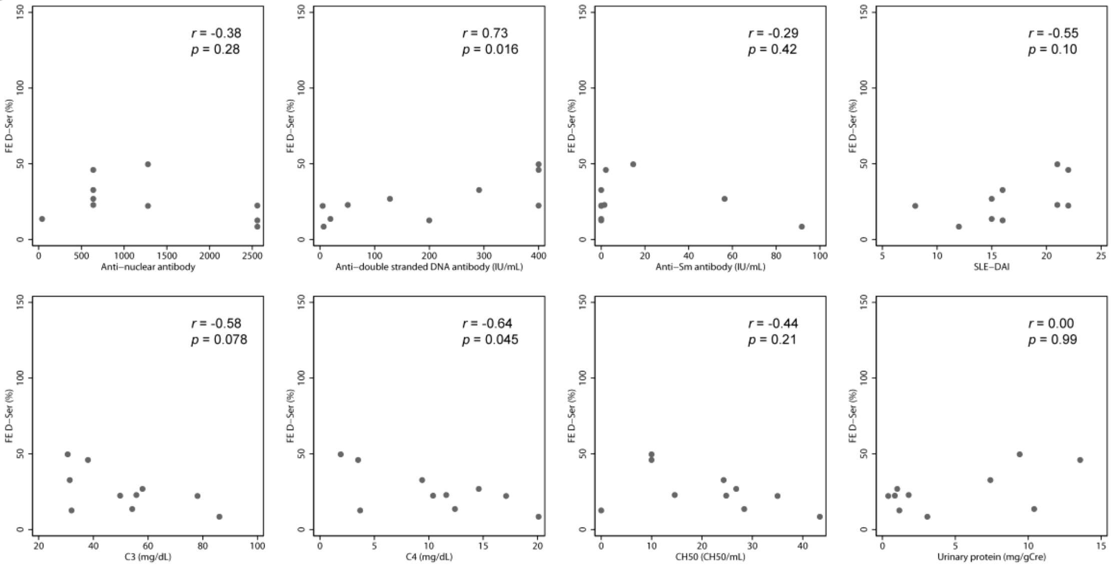

c

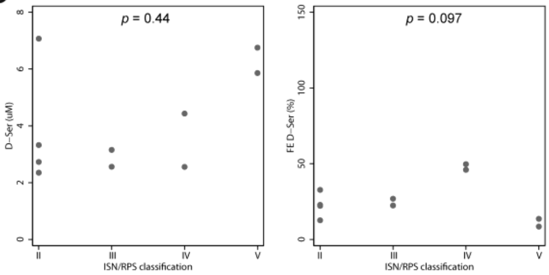

Supplementary Fig. 3 Relation of D-serine profile and activity of lupus nephritis. a, b Relation between (**a**) plasma level of D-serine profile, and (**b**) fractional excretion (FE) of D-serine, with clinical and laboratory data that are associated with SLE or reflect its activity. **c** Profile of D-serine in histological categories of LN based on the 2003 International Society of Nephrology / Renal Pathology Society classification. SLE-DAI, SLE Disease Activity Index.

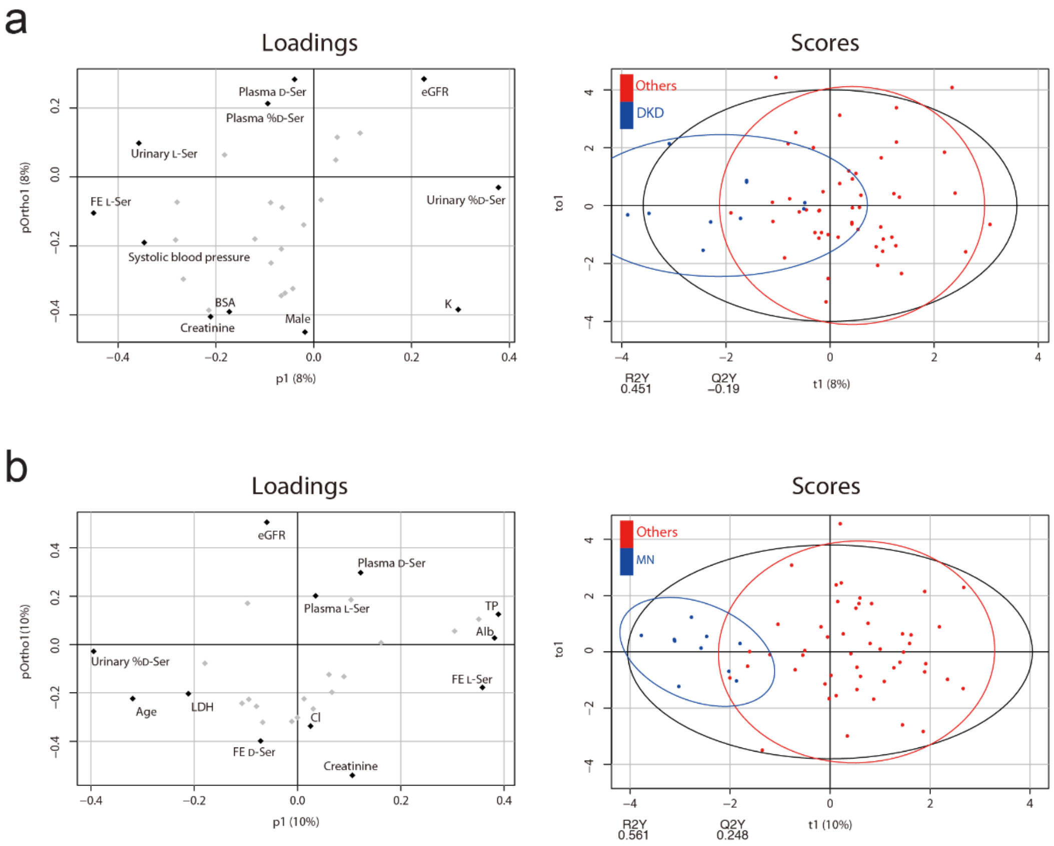

Supplementary Fig. 4 Relation of D-serine profile in the separation of kidney diseases. a, **b** Orthogonal projection to latent structure-discriminant analysis (OPLS-DA) plots in (**a**) DN and (**b**) MN patients compared with the rest of studied population. FE, fractional excretion; .eGFR, estimated glomerular filtration rate; BSA, body surface area; TP, total protein; Alb, albumin.

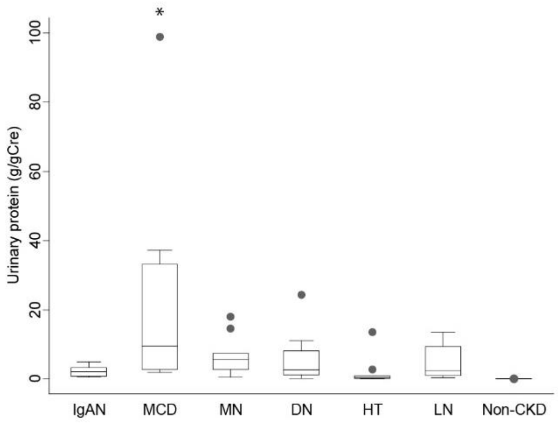

Supplementary Fig. 5 Urinary level of protein in various kidney diseases. IgAN, IgA nephritis; MCD, minimal change disease; MN, membranous nephropathy; DN, diabetic nephropathy; HT, hypertensive nephropathy; LN, lupus nephritis; CKD, chronic kidney disease. Reference data of non-CKD are from [7]. *n* = 10 for each disease and 15 for non-CKD. *$P < 0.05$ versus non-CKD (one-way ANOVA).

Table S1. Baseline characteristics of each kidney disease.

	IgAN (n = 10)	MCD (n = 10)	MN (n = 10)	DN (n = 10)	HT (n = 10)	LN (n = 10)
Age, yr	35 (30 - 41)	53 (43 - 66)	72 (63 - 79)	55 (45 - 66)	60 (55 - 63)	42 (39 - 52)
Male gender, %	90 (9)	50 (5)	90 (9)	60 (6)	90 (9)	0 (0)
Body mass index, kg/m^2	23.4 (21.2 - 25.8)	26.0 (23.9 - 27.0)	25.4 (24.1 - 26.6)	26.9 (26.5 - 28.0)	23.5 (22.8 - 27.2)	20.3 (18.7 - 22.6)
Systolic blood pressure, mmHg	125 (113 - 132)	129 (123 - 137)	153 (137 - 155)	162 (149 - 165)	138 (129 - 158)	124 (107 - 138)
Diastolic blood pressure, mmHg	73 (72 - 79)	81 (72 - 86)	87 (75 - 92)	87 (81 - 95)	90 (75 - 108)	80 (69 - 84)
Serum protein, g/dL	7.1 (6.5 - 7.3)	4.9 (4.5 - 5.4)	5.0 (4.6 - 5.4)	5.9 (5.3 - 6.7)	7.2 (6.7 - 7.6)	6.4 (5.7 - 6.8)
Serum albumin, g/dL	4.3 (4.1 - 4.4)	2.0 (1.6 - 2.1)	2.0 (1.9 - 2.5)	3.0 (2.4 - 3.6)	4.4 (4.1 - 4.5)	2.7 (2.4 - 2.8)
Serum Creatinine, mg/dL	1.01 (0.98 - 1.04)	0.83 (0.67 - 1.00)	0.84 (0.75 - 0.87)	1.14 (1.10 - 1.19)	1.13 (0.87 - 1.44)	0.66 (0.62 - 0.78)
eGFR $_{creat}$, mL/min/1.73m^2	67.8 (63.8 - 72.2)	62.6 (55.1 - 82.8)	67.2 (64.0 - 72.4)	45.8 (39.6 - 55.8)	52.2 (38.8 - 70.9)	78.2 (61.6 - 87.1)
Serum UN, mg/dL	13.0 (12.0 - 14.0)	12.0 (10.3 - 17.0)	17.0 (14.0 - 19.5)	14.0 (12.5 - 14.8)	15.5 (14.3 - 18.8)	9.0 (7.3 - 14.5)
Serum Na, mEq/L	141 (139 - 142)	139 (138 - 139)	140 (139 - 141)	141 (139 - 141)	140 (138 - 141)	136 (134 - 138)
Serum K, mEq/L	4.1 (4.1 - 4.3)	4.3 (4.1 - 4.7)	4.4 (4.0 - 4.5)	3.9 (3.6 - 4.3)	4.1 (3.9 - 4.4)	4.1 (3.8 - 4.5)
Serum Cl, mEq/L	106 (105 - 106)	105 (104 - 107)	106 (103 - 108)	107 (104 - 107)	106 (105 - 107)	102 (100 - 104)
ALT, U/L	22 (19 - 26)	21 (15 - 35)	15 (12 - 22)	22 (16 - 27)	19 (14 - 39)	23 (15 - 30)
LDH, U/L	198 (162 - 229)	230 (207 - 246)	251 (225 - 276)	226 (210 - 283)	171 (156 - 200)	246 (215 - 298)
Urinary protein, g/gCre	2.11 (0.81 - 3.19)	9.50 (3.07 - 29.30)	5.70 (3.14 - 7.11)	2.69 (1.28 - 7.33)	0.34 (0.14 - 0.76)	2.44 (1.06 - 8.92)
Urinary NAG, IU/gCre	16.1 (5.2 - 22.6)	17.9 (5.9 - 45.9)	10.8 (5.7 - 23.1)	22.6 (12.4 - 42.9)	9.6 (7.4 - 18.8)	21.9 (9.0 - 28.1)
Urinary β2-MG, mg/gCre	0.43 (0.23 - 1.10)	0.18 (0.08 - 0.94)	0.12 (0.05 - 0.19)	0.66 (0.05 - 2.12)	0.59 (0.09 - 5.62)	0.37 (0.19 - 1.40)
Hypertension history, %	0 (0)	50 (5)	80 (8)	90 (9)	100(10)	30 (3)
Diabetes history, %	0 (0)	0 (0)	20 (2)	100 (10)	10(1)	0 (0)
Hyperlipidemia history, %	0 (0)	20 (2)	60 (6)	60 (6)	10(1)	0 (0)
Use of RASI, %	0 (0)	20 (2)	60 (6)	90 (9)	40 (4)	10 (1)
use of other antihypertensive drug, %	0 (0)	20 (2)	60 (6)	80 (8)	50 (5)	0 (0)
Use of diuretics, %	0 (0)	30 (3)	70 (7)	40 (4)	20 (2)	30 (3)

Values are described as median (IQR) or % (count). RASI, renin-angiotensin system inhibitors.
